# Supplementary material for: Predicting cell type-specific epigenomic profiles accounting for distal genetic effects
Source: Nat Commun. 2024 Nov 16;15:9951. doi: 10.1038/s41467-024-54441-5 (PMC11569248; doi:10.1038/s41467-024-54441-5)
Supplement: Supplementary file 1 — Supplementary Information [file 41467_2024_54441_MOESM1_ESM.pdf]

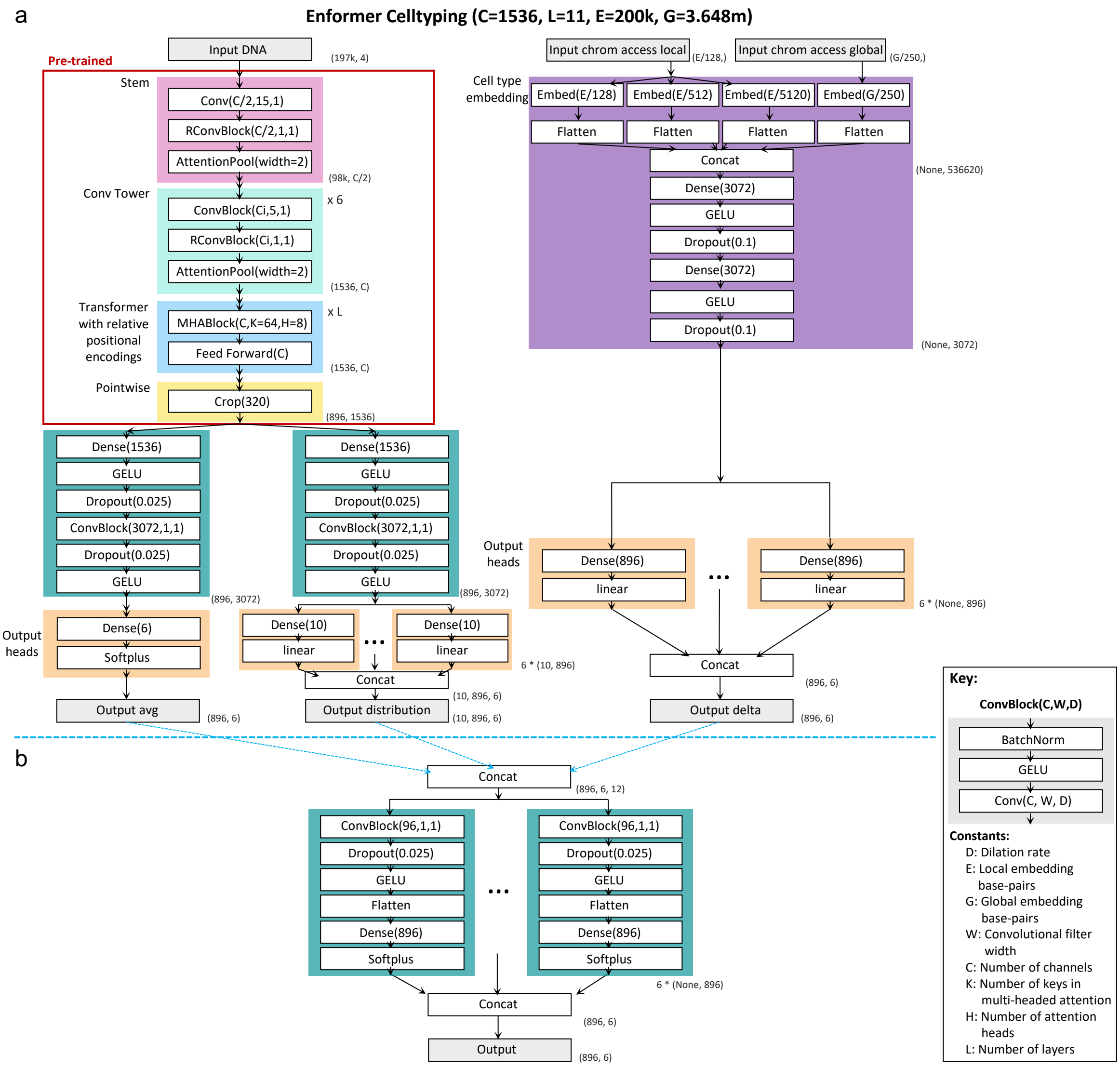

Fig. 1 Enformer Celltyping architecture. (a) pre-training, warm-up step: Enformer Celltyping's architecture is split into two separate submodules – The DNA half (stemming from 'Input DNA') which inputs DNA sequence and uses a pre-trained, chopped, frozen version of Enformer through a transfer learning approach to predict an average histone mark signal ('Output avg') and distribution ('Output distribution') across training cell types for the genomic region; and the celltyping half (stemming from 'Input chrom access local' and 'Input chrom access global') which embeds chromatin accessibility data from the same region and predicts the difference between the average histone mark signal and the cell type-specific signal ('Output delta'). (b) Second training step: Enformer Celltyping combines the three outputs

(‘Output avg’,‘Output distribution’ and ‘Output delta’) with subsequent layers to predict the cell type-specific histone mark signal for a given genomic region.

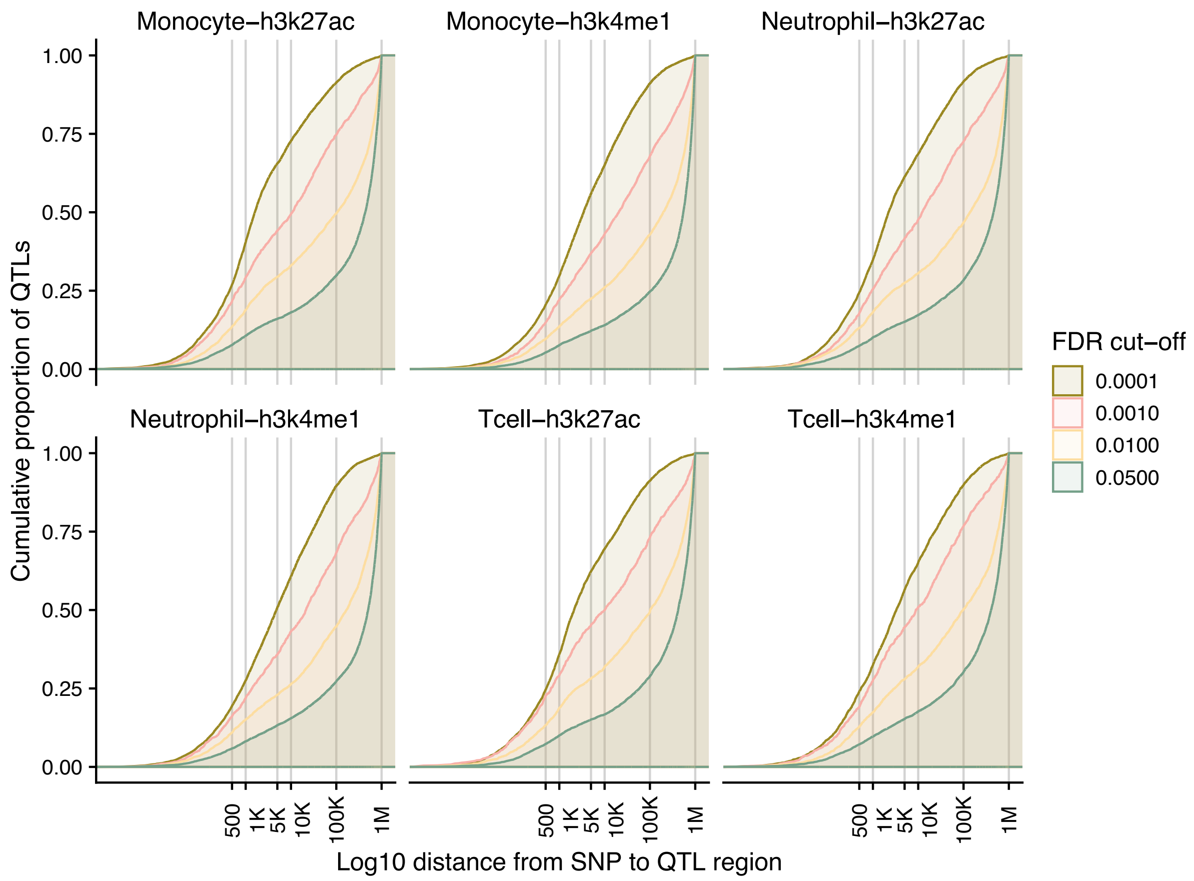

Fig. 2 Histone mark QTL data shows that SNPs regulate peaks that are significant distances away. The distance from the SNP to the associated histone mark peak for the six histone mark quantative trait loci (hQTL) studies from BLUEPRINT Phase 2 are shown at increasing FDR cut-offs. Here, to avoid double counting due to linkage disequilibrium (LD), SNPs were aggregated to those with the lowest p-value for each LD block based on Pickrell LD blocks. The y-axis is the cumulative proportion of interactions and the x-axis is the $\log_{10}$ distance with a grey vertical lines increasing numbers of base-pairs. At all FDR cut-offs, for all hQTL studies, we observe high proportions of interactions (between 20%-75%) at distances above 10,000 base-pairs. This goes against a null hypothesis that SNPs only regulate the epigenome by local motif disruption and highlights the need for models which can account for these distal interactions when predicting epigenetic profiles. Source data are provided as a Source Data file.

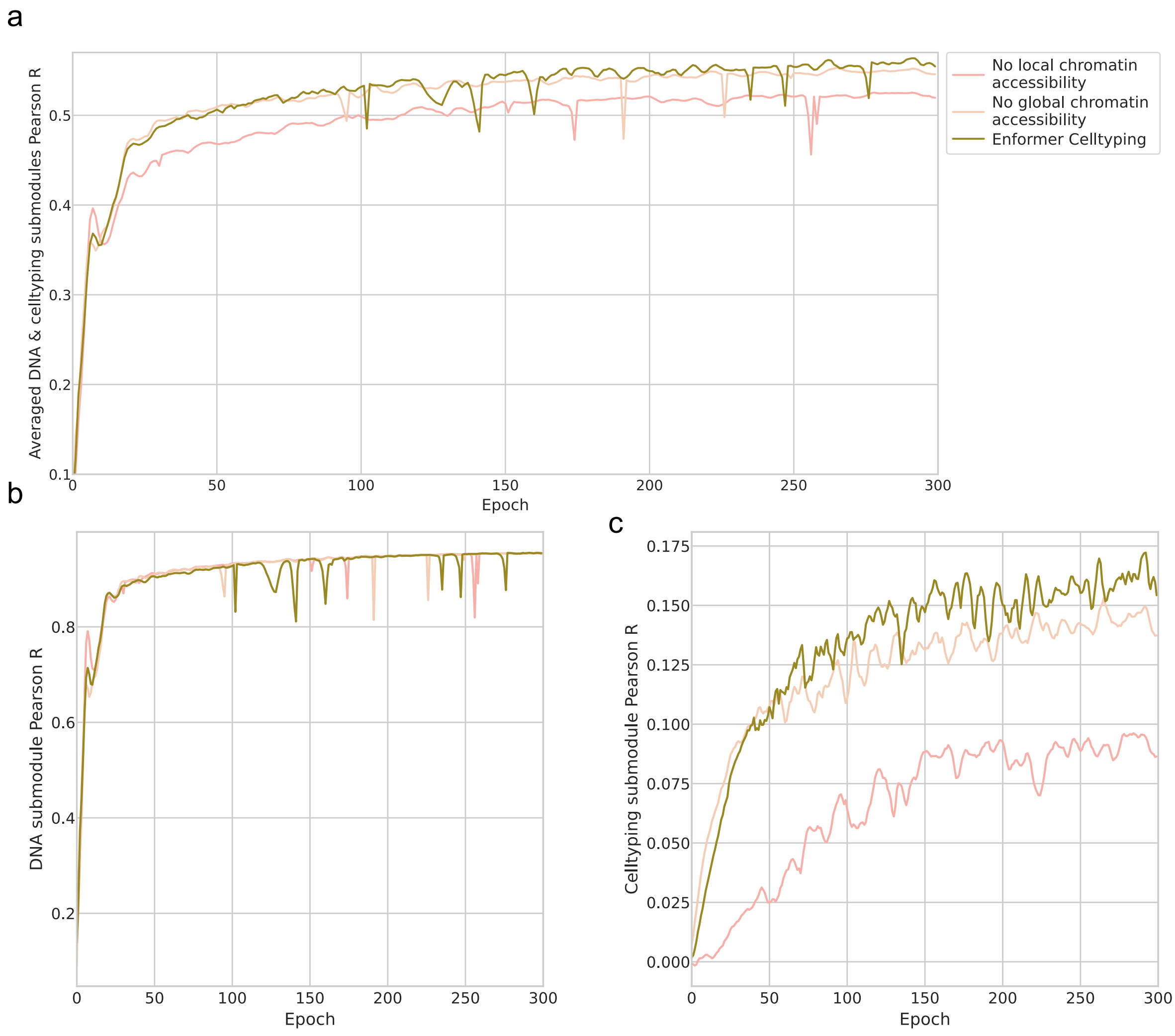

Fig. 3 Using global and local chromatin accessibility information improves the prediction of cell type-specific histone mark signals. We measured the performance of Enformer Celltyping on the validation set for the pre-training stage over 300 training steps (batch size 128) for both global and local chromatin accessibility information (green), no local chromatin accessibility (pink) and no global chromatin accessibility (beige). (a) Averaged Pearson R performance for both the DNA and celltyping submodules, (b) DNA submodule – note this is a negative control, these should be the same since the chromatin accessibility only effects the celltyping submodule and (c) the celltyping submodule – where this should have the largest effect. All Pearson R values are performance on the validation dataset. Source data are provided as a Source Data file.

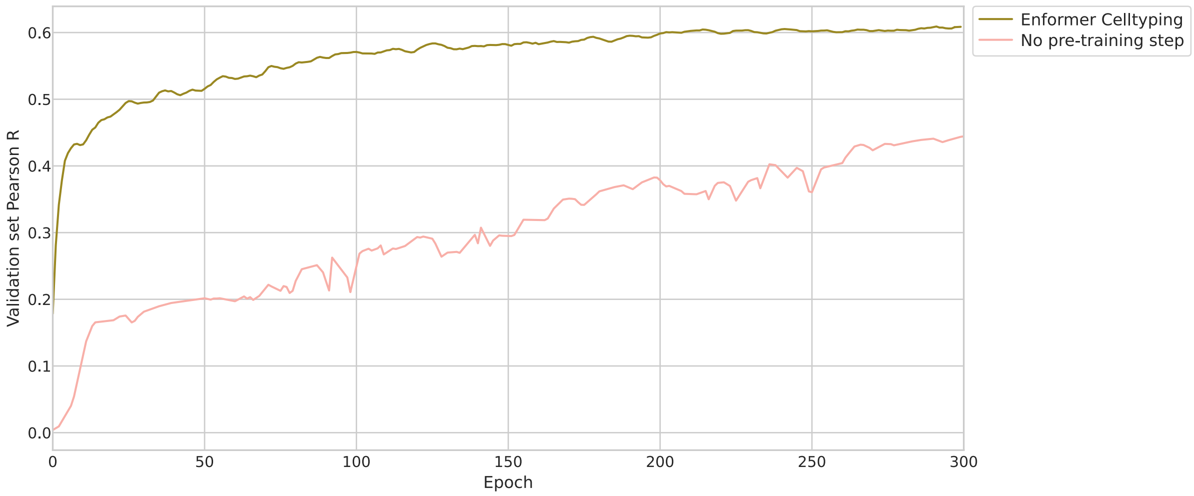

Fig. 4 Pre-training drastically improves Enformer Celltyping's performance. We measured the performance of Enformer Celltyping on the validation set for the full training stage over 300 training steps (batch size 128) comparing the full Enformer Celltyping model (green) against a version of Enformer Celltyping for which a pre-training stage was not performed (pink). Pearson R values are performance on the validation dataset. Source data are provided as a Source Data file.

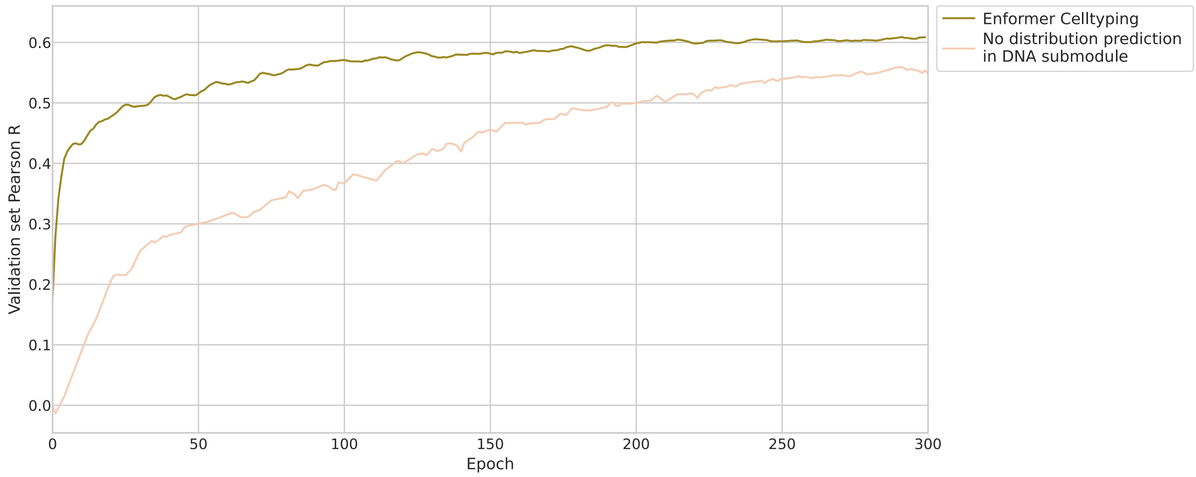

Fig. 5 Predicting the distribution of signals as well as the average with the DNA module during pre-training improves Enformer Celltyping's performance. We measured the performance of Enformer Celltyping on the validation set for the full training stage over 300 training steps (batch size 128) comparing the full Enformer Celltyping model (green) against a version of Enformer Celltyping for which the DNA module only predicted the average expression, as opposed to the average and distribution, in the pre-training stage (beige). Pearson R values are performance on the validation dataset. Source data are provided as a Source Data file.

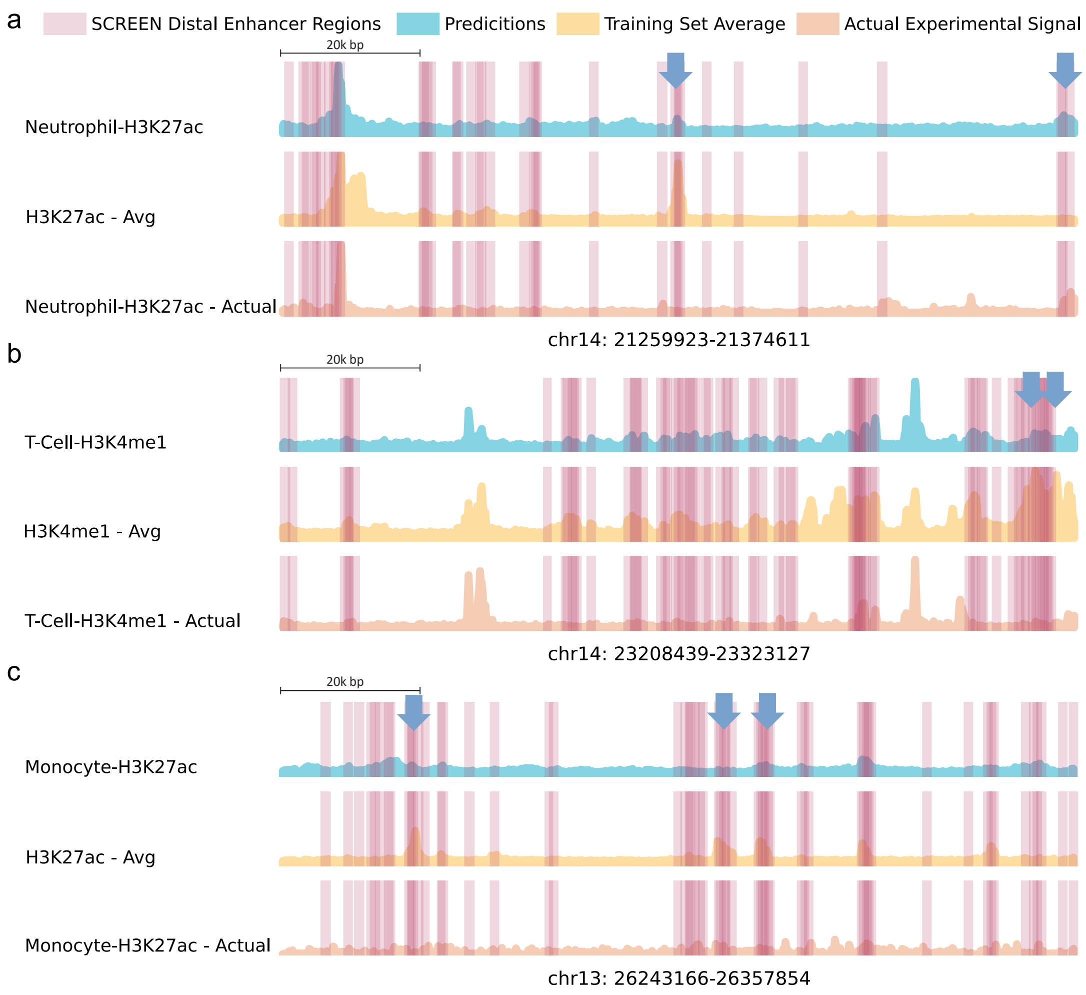

Fig. 6 Enformer Celltyping predicts cell type-specific histone marks signals that match the experimental track. Enformer Celltyping's H3K27ac (a,c) and H3K4me1 (b) predictions (blue), the training set average (gold) and the experimental assay signals (apricot) at differing genomic loci with SCREEN distal enhancer regions highlighted for the three blood immune cells. Navy arrows indicate true peaks in SCREEN distal enhancer regions either missed by the average but captured by Enformer Celltyping or not in the experiment or Enformer Celltyping but captured by the average. We can see Enformer Celltyping does not simply predict the average and outperforms it, managing to capture cell type-specific signals for previously

unseen cell types. chr2:136,741,919-136,926,747. Source data are provided as a Source Data file.

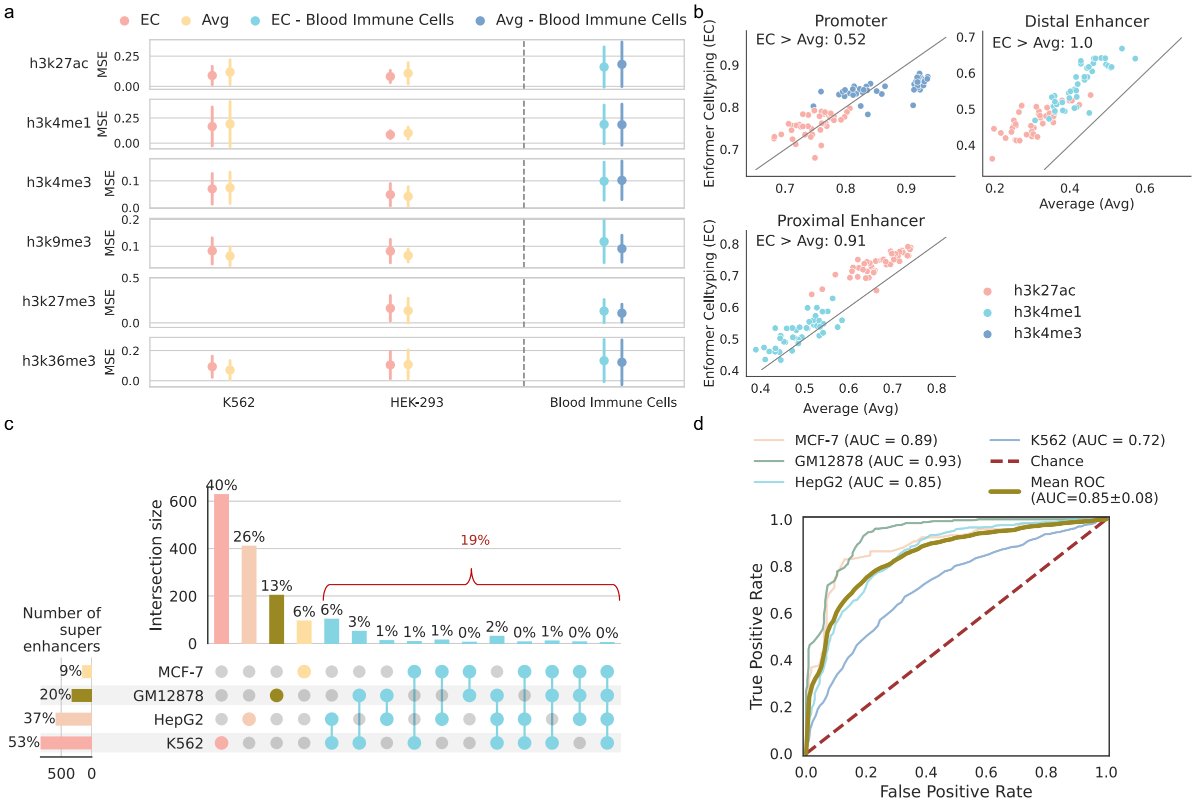

Fig. 7 Enformer Celltyping shows superior performance on cancer cell lines and its embeddings can discriminate super-enhancers. (a) Enformer Celltyping's (EC) mean squared error (MSE) across two held-out ENCODE cancer cell lines. The average model (Avg) performance and the average performance across the three ENCODE immune cell types (Blood Immune Cells) is shown for comparison. The results for the "Blood immune cells" is the union of prediction results for T-cells, Neutrophils and Monocytes. "Average" here refers to the MSE calculated using the average values for the histone mark, across the training cell types. Performance for H3K27me3 in K562 is not shown as it has not been assayed. Performance is based on genome-wide predictions (over 16,000 per cell type) with error bars for the standard deviation. (b) EC (y-axis) versus Avg (x-axis) Pearson R correlation performance with the experimental signal in functional genomic regions from Search Candidate cis-Regulatory Elements by ENCODE (SCREEN). Performance was measured for histone marks with known functional roles in these regions for the two cancer cell lines (K562 and HEK-293). Each point represents the Pearson R performance for each against the true signal for each cell type-

chromosome combination. The EC > Avg represents for how many cell type-chromosome values our model outperformed the average. (c) An upset plot showing the overlap of super-enhancers (SEs) across four ENCODE cancer cell lines, highlighting the cell type-specificity of the SEs, with only 19% having any overlap across the cells. (d) Performance on held-out cells for random forest classifiers trained on features derived from Enformer Celltyping, to discriminate super-enhancers from all enhancers. Source data are provided as a Source Data file.

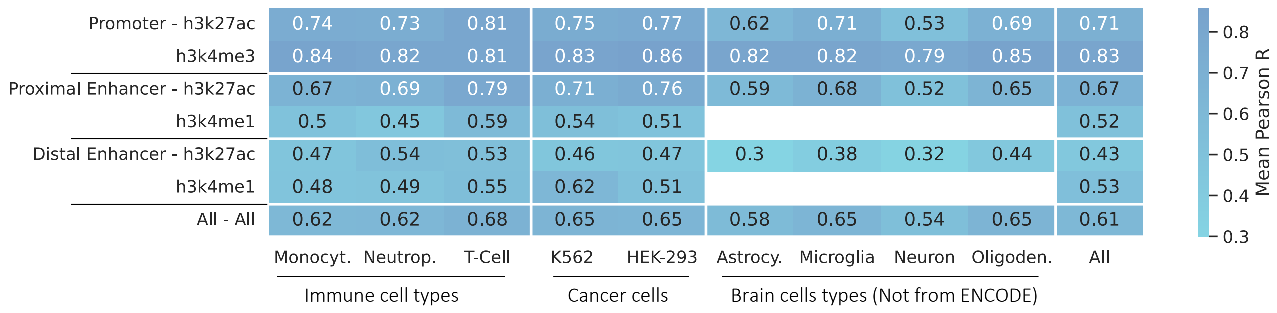

Fig. 8 Enformer Celltyping's performance remains consistent across diverse cell types. Enformer Celltyping's Pearson R correlation performance with the experimental signal in functional genomic regions from Search Candidate cis-Regulatory Elements by ENCODE (SCREEN) (rows) across the 9 diverse cell types tested (columns). This performance is shown for the functionally relevant histone marks and the cell types are split into broad tissue groups. Monocyt. is Monocyte, Neutrop. is Neutrophil, Astrocy. is Astrocyte and Oligoden. is Oligodendrocyte. The weighted average of the three SCREEN regions for the histone marks for each cell type (last row) and for each SCREEN and histone mark combination (last column) is shown. We can see that on average, Astrocytes had the worst performance which is consistent with the results in Fig. 5b and is likely attributable to the low sequencing depth of the associated ATAC-Seq data. Importantly however, average performance for cell types was approximately a Pearson R of 0.65, even on cells from outside of ENCODE (Microglia and Oligodendrocytes), highlighting Enformer Celltyping's consistent performance across cell types. Overall, performance dropped from proximal to distal regions for H3K27ac (0.67 to 0.43) but not H3K4me1 (0.52 to 0.53). Source data are provided as a Source Data file.

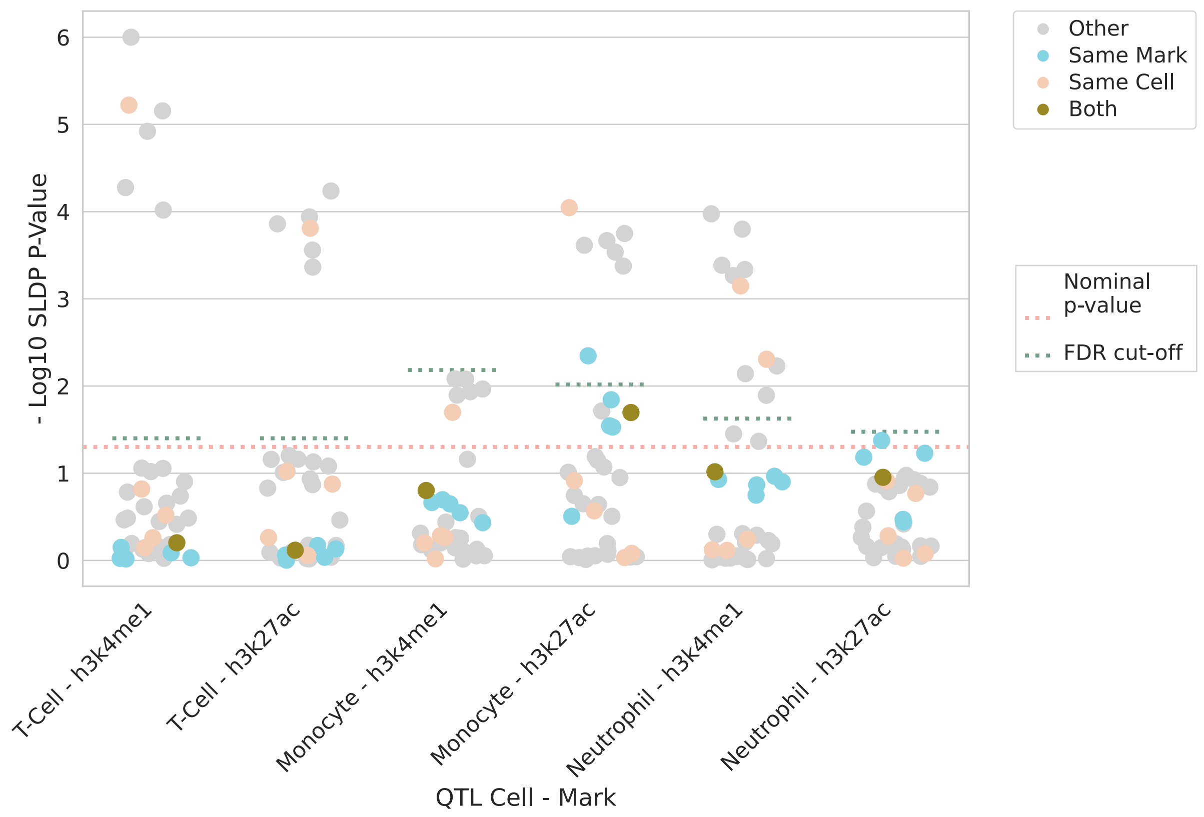

Fig. 9 Enformer Celltyping's genetic effect predictions are not noticeably improved by filtering out QTLs associated with downregulating peaks which are missed in predictions for the major allele. Statistical significance (y-axis) of SLDP genome-wide concordance between Enformer Celltyping's genetic variant predictions and measured hQTL effect sizes. Here, any SNP to histone mark binding hQTL entries were removed if it caused a decrease in binding and Enformer Celltyping did not predict the binding on the major allele (on the DNA sequence without the genetic variant). The x-axis depicts the six Blueprint phase 2 hQTL studies and the cell-histone mark predictions are coloured by their relationship to the hQTL study: 'Both' – both the histone mark and the cell predicted in were the same as the hQTL study, 'Same Mark' – the mark matched, 'Same Cell' – the cell matched and 'Other' – neither the cell nor mark matched. If there was perfect agreement between our model and the hQTL studies, all 'Both' entries would be significant. A nominal p-value (pink dashed line) and the cut-off after Benjamini and Hochberg correction based on the minimum, non-significant p-

value, corrected at the level of each hQTL (Turquoise dashed line) are both added. Source data are provided as a Source Data file.

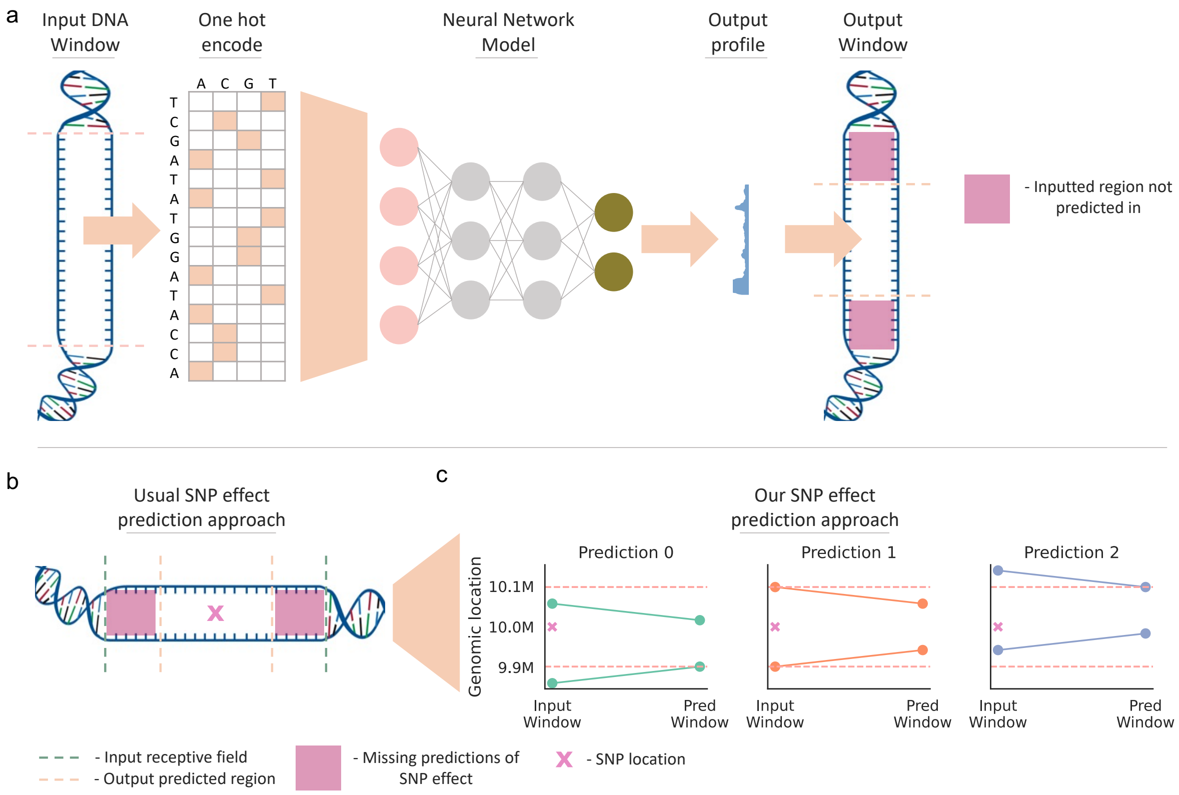

Fig. 10 Schematic showing our strategy to capture distal effect of SNPs, by predicting each SNP multiple times, sliding the input window around the SNP location. Genomic deep learning models tend to predict profiles for only the centre proportion of the genomic location relating to the DNA input to avoid predicting on edge regions which have less neighbouring genomic information. This funnel effect is shown in (a). Thereafter, common practice when predicting the effect of SNPs (i.e. *in silico* mutagenesis) with these models, is to centre the SNP on the input window (b). However, this approach misses distal predictions of SNP effects, not using the full receptive field of the model. We propose an alternative strategy to capture the effect of the SNP across the full receptive field by predicting each SNP multiple times, sliding the input window around the SNP location, e.g. move the relative SNP location towards the edges, or closer to the centre (c). For Enformer Celltyping, this requires three predictions (c): A prediction made with the SNP in the centre of the input window (‘Prediction 1’) to capture the nearby effect of the SNP and sliding the input window to make two further predictions (‘Prediction 0’ and ‘Prediction 2’) to capture distal SNP predictions.

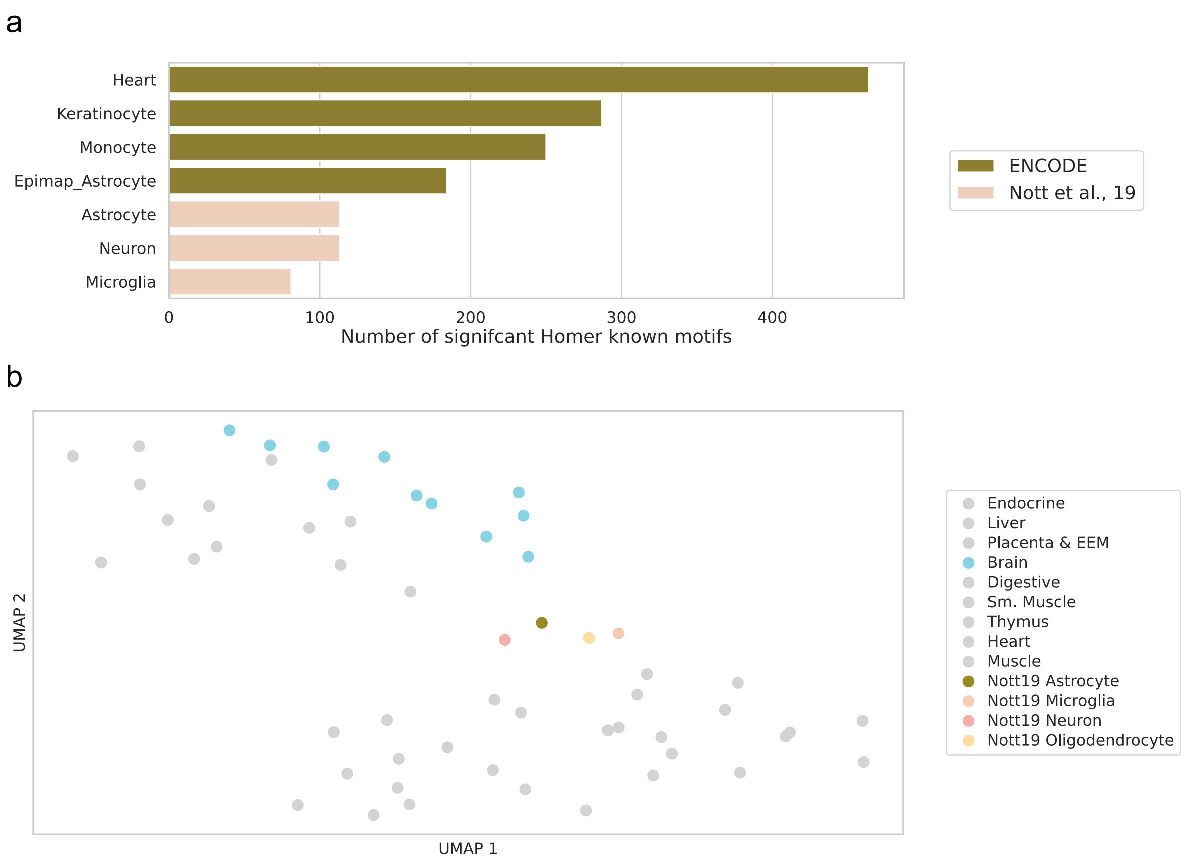

Fig. 11 Enformer Celltyping's embedded global chromatin accessibility signal appears biased towards ENCODE samples. (a) Gives the number of significant known motifs identified with Homer for each of the six cell types tested. The genomic regions for the motif analysis were derived from the histone mark peaks that had the highest contribution from the global chromatin accessibility signal for each cell type, identified by dropping the global signal inputted to the model and measuring the change in predicted binding. The cell types are coloured by their source. (b) A UMAP projection of Enformer Celltyping's global chromatin accessibility embeddings. Both training cell types and the four isolated cortical brain cell types from Nott et al. are included (the later are coloured). The other training cell types related to the brain (all bulk tissue, not isolated cells) are also coloured. Source data are provided as a Source Data file.

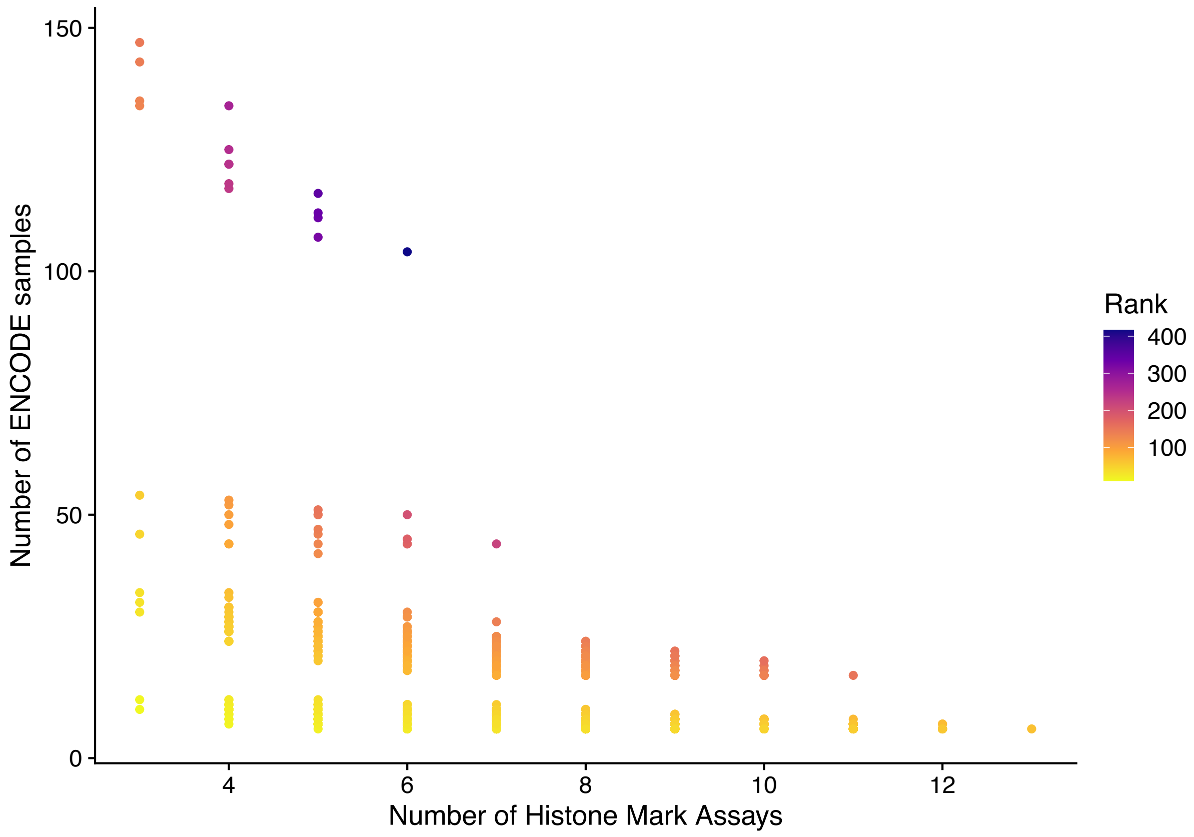

Fig. 12 EpiMap trade-off between the number of samples and the number of assays. The histone mark data available from EpiMap is shown with the number of samples (cell/tissue type) along the y-axis at each of the different number of histone mark assays along the x-axis. When choosing the number of assays to include in the model's predictions, the aim was to maximise both the number of assays but also the number of samples. The colour, which is the product of these numbers, of each position gives a rank which takes both into account. Note that the number of samples exclude our 3 validation cells (CD14+ monocytes, CD16+ neutrophils and naive CD4+T cells) and any related cell types. Source data are provided as a Source Data file.

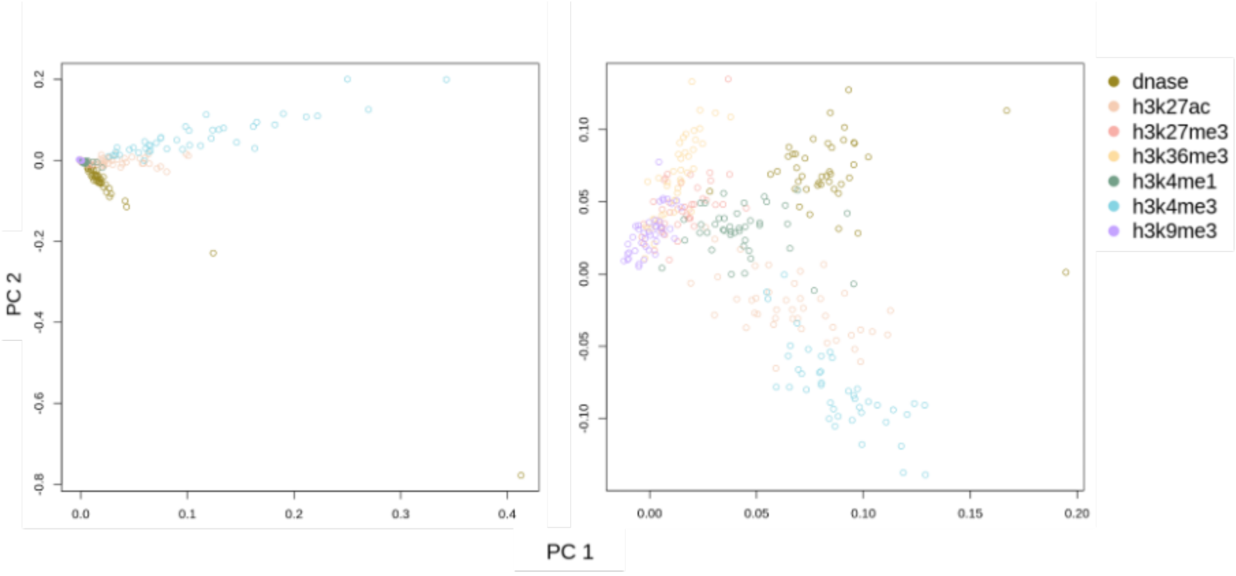

Fig. 13 Arcsinh-transformed epigenetic signals for same marks show groupings. Principal component analysis (PCA) plot of the first two principal components without (left) and with (right) arcsinh-transform based on the EpiMap training samples. Note that the data is not scaled here (a usual step in PCA) to replicate the data a model would be trained on. The arcsinh-transformed data shows clear separation between assay types, something which is not present with the raw data, highlighting arcsinh-transform's benefit. Note that DNase-Seq assayed were included here to show chromatin accessibility differentiation although they were not used in the model training approach. Source data are provided as a Source Data file.

Group	Secondary	Type	BSSID	Extended Info
Cancer	Lung	cell line	BSS00013	LUNG EPITHELIAL CARCINOMA
Cancer	Lung	cell line	BSS00015	LUNG EPITHELIAL CARCINOMA
Cancer	Muscle	cell line	BSS00035	MUSCLE EWING SARCOMA
Endocrine	NA	tissue	BSS00060	ADRENAL GLAND
Brain	NA	tissue	BSS00078	ANGULAR GYRUS
Heart	NA	tissue	BSS00088	ASCENDING AORTA
Brain	NA	primary cell	BSS00089	ASTROCYTE
Other	NA	tissue	BSS00146	BREAST EPITHELIUM
ES-deriv	Heart	in vitro differentiated cells	BSS00171	CARDIAC MUSCLE DERIV
Brain	NA	tissue	BSS00173	CAUDATE NUCLEUS
Brain	NA	tissue	BSS00175	CAUDATE NUCLEUS
Placenta & EEM	NA	tissue	BSS00214	CHORIONIC VILLUS
Placenta & EEM	NA	tissue	BSS00215	CHORIONIC VILLUS
Placenta & EEM	NA	tissue	BSS00216	CHORIONIC VILLUS
Placenta & EEM	NA	tissue	BSS00217	CHORIONIC VILLUS
Brain	NA	tissue	BSS00220	CINGULATE GYRUS
Digestive	NA	tissue	BSS00227	COLON MUCOSA
Digestive	NA	tissue	BSS00228	COLON MUCOSA
HSC & B-cell	NA	primary cell	BSS00231	CD34 CMP
HSC & B-cell	NA	primary cell	BSS00232	CD34 CMP
Cancer	Blood & T-cell	cell line	BSS00267	ACUTE LYMPHOBLASTIC LEUKEMIA
Digestive	NA	tissue	BSS00270	DUODENUM MUCOSA
ES-deriv	NA	in vitro differentiated cells	BSS00273	ECTODERMAL DERIV
ES-deriv	NA	in vitro differentiated cells	BSS00287	ENDODERMAL DERIV
Endothelial	Placenta & EEM	primary cell	BSS00296	UMBILICAL VEIN ENDOTHELIAL CELL
Digestive	NA	tissue	BSS00316	ESOPHAGUS
Digestive	NA	tissue	BSS00318	ESOPHAGUS
Stromal	Lung	primary cell	BSS00341	LUNG FIBROBLAST
Stromal	NA	primary cell	BSS00353	FORESKIN FIBROBLAST
Epithelial	NA	primary cell	BSS00354	FORESKIN KERATINOCYTE
Other	NA	primary cell	BSS00368	FORESKIN MELANOCYTE
Muscle	NA	tissue	BSS00379	GASTROCNEMIUS MEDIALIS
Lymphoblastoid	NA	cell line	BSS00439	LYMPHOBLASTOID CELL LINE
Stromal	NA	cell line	BSS00476	SKIN FIBROBLAST
ESC	NA	cell line	BSS00478	ESC
ESC	NA	cell line	BSS00484	ESC
Cancer	Digestive	cell line	BSS00492	COLORECTAL ADENOCARCINOMA
Heart	NA	tissue	BSS00507	HEART LEFT VENTRICLE
Heart	NA	tissue	BSS00512	HEART LEFT VENTRICLE
Heart	NA	tissue	BSS00513	HEART LEFT VENTRICLE
Heart	NA	tissue	BSS00525	HEART RIGHT VENTRICLE
Cancer	Reproductive	cell line	BSS00529	CERVIX ADENOCARCINOMA
Cancer	Liver	cell line	BSS00558	HEPATOCELLULAR CARCINOMA
ESC	NA	cell line	BSS00715	ESC
ESC	NA	cell line	BSS00716	ESC
ESC	NA	cell line	BSS00717	ESC
Stromal	Lung	cell line	BSS00720	LUNG FIBROBLAST
iPSC	NA	cell line	BSS00731	iPSC
iPSC	NA	cell line	BSS00737	iPSC
iPSC	NA	cell line	BSS00739	iPSC
Cancer	HSC & B-cell	cell line	BSS01065	B CELL LYMPHOMA
Epithelial	NA	primary cell	BSS01068	KERATINOCYTE
Digestive	NA	tissue	BSS01119	LARGE INTESTINE
Brain	NA	tissue	BSS01124	HIPPOCAMPUS
Brain	NA	tissue	BSS01125	HIPPOCAMPUS
Brain	NA	tissue	BSS01126	HIPPOCAMPUS
Liver	NA	tissue	BSS01159	LIVER
Liver	NA	tissue	BSS01168	LIVER
Lung	NA	tissue	BSS01190	LUNG
Epithelial	NA	primary cell	BSS01213	MAMMARY EPITHELIAL CELL
Cancer	Other	cell line	BSS01226	ADENOCARCINOMA
Brain	NA	tissue	BSS01272	MIDDLE FRONTAL AREA
Cancer	HSC & B-cell	cell line	BSS01274	MYELOMA
Digestive	NA	tissue	BSS01282	RECTUM MUCOSA
Digestive	NA	tissue	BSS01283	RECTUM MUCOSA
Sm. Muscle	Digestive	tissue	BSS01287	DUODENUM MUSCLE
Muscle	NA	tissue	BSS01319	LEG MUSCLE
Muscle	NA	tissue	BSS01332	TRUNK MUSCLE
Myosat	NA	in vitro differentiated cells	BSS01344	MYOTUBE
ES-deriv	Brain	in vitro differentiated cells	BSS01371	NEURAL PROGENITOR DERIV
Neurosph	NA	primary cell	BSS01377	NEUROSPHERE
Cancer	HSC & B-cell	cell line	BSS01390	B CELL LYMPHOMA
Bone	NA	primary cell	BSS01397	OSTEOBLAST
Endocrine	Reproductive	tissue	BSS01399	OVARY

Cancer	Pancreas	cell line	BSS01405	PANCREAS DUCT EPITHELIAL CARCINOMA
Pancreas	NA	tissue	BSS01406	PANCREAS
Cancer	Reproductive	cell line	BSS01414	PROSTATE ADENOCARCINOMA
Cancer	Lung	cell line	BSS01415	LUNG ADENOCARCINOMA
Blood & T-cell	NA	primary cell	BSS01423	MONONUCLEAR CELL
Placenta & EEM	NA	tissue	BSS01431	PLACENTA
Placenta & EEM	NA	tissue	BSS01438	PLACENTA
Placenta & EEM	NA	tissue	BSS01441	PLACENTA
Placenta & EEM	NA	tissue	BSS01446	PLACENTA
Muscle	NA	tissue	BSS01463	PSOAS MUSCLE
Digestive	NA	tissue	BSS01545	SIGMOID COLON
Digestive	NA	tissue	BSS01548	SIGMOID COLON
Cancer	Brain	cell line	BSS01562	NEUROBLASTOMA
Myosat	NA	primary cell	BSS01574	SKELETAL MUSCLE MYOBLAST
Muscle	NA	tissue	BSS01578	SKELETAL MUSCLE
Digestive	NA	tissue	BSS01588	SMALL INTESTINE
Digestive	NA	tissue	BSS01601	SMALL INTESTINE
ES-deriv	Sm. Muscle	in vitro differentiated cells	BSS01612	SMOOTH MUSCLE DERIV
Spleen	NA	tissue	BSS01631	SPLEEN
Digestive	NA	tissue	BSS01639	STOMACH
Digestive	NA	tissue	BSS01651	STOMACH
Sm. Muscle	Digestive	tissue	BSS01659	STOMACH MUSCLE
Adipose	NA	tissue	BSS01667	ADIPOSE TISSUE
Brain	NA	tissue	BSS01676	SUBSTANTIA NIGRA
Brain	NA	tissue	BSS01712	TEMPORAL LOBE
Brain	NA	tissue	BSS01714	TEMPORAL LOBE
Heart	NA	tissue	BSS01814	THORACIC AORTA
Thymus	NA	tissue	BSS01820	THYMUS
Thymus	NA	tissue	BSS01825	THYMUS
ES-deriv	Placenta & EEM	in vitro differentiated cells	BSS01857	TROPHOBLAST DERIV

Table 1 Training samples and EpiMap IDs

Why predict -$\log_{10}$ p-values over fold change from MACS 2 for ChIP-Seq histone mark data with Enformer Celltyping?

We agree that using the fold change output from ENCODE's MACS2 processing pipeline (log2fc) has its benefits and has been used by other models[1,2]. Equivalently many models, especially those imputing epigenetic signals in previously unseen cell types, use the -$\log_{10}$ p-value signal[3–5]. Moreover, when considering the differences between the two, the -$\log_{10}$ p-value was found to have better signal-to-noise properties and so, was thought to be more suitable. For this reason, ENCODE chose -$\log_{10}$ p-value as the primary signal tracks for their analyses[6]. This is discussed in more detail by Ernst and Kellis[7]:

"We selected the $-\log_{10}$ P value signal tracks rather than the fold-change tracks for histone marks and DNase as they were designated the primary signal tracks for analyses in (Roadmap Epigenomics Consortium et al., 2015) on the basis of having better signal-to-noise properties. In particular, both sets of tracks were generated based on down sampling highly sequenced datasets to the same sequencing depth, thus in the $-\log_{10}$ P value track, no dataset had a disproportionately high signal simply due to being sequenced very deeply, whereas on the other hand under-sequenced datasets were included and in some cases had locations with high fold-change signals that were the result of noise and did not have values as relatively high on the $-\log_{10}$ P-value track."

We hope, based on this, it is clear why we chose to model the -$\log_{10}$ p-value with Enformer Celltyping especially since we are considering predictions across cell types (like previous imputation approaches). Moreover, -$\log_{10}$ p-values are more interpretable which is useful, for example, thresholding for peaks such as what ChromHMM does[4]. This can't be easily done with logfc values.

In our work, we also went a step further in addressing the "disproportionately high signals" in some genomic locations that were less common but still present in the $-\log_{10}$ P-value. We processed the p-value signal using an arc-sinh transformation to help with differences in sequencing depth as others have done[3,5]. The arc-sinh transformation 'tows in' extreme values so these do not affect the model training, see Figure below:

$$\sinh^{-1} x = ln\left(x + \sqrt{1 + x^2}\right)$$

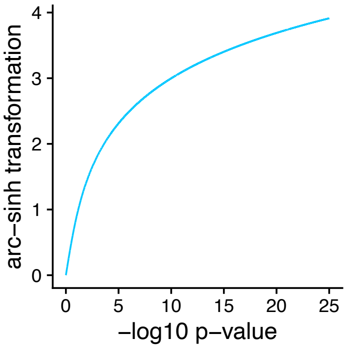

Also please see Fig. s11 in the supplementary of the manuscript (Additional file 1: Fig. S11) where we show the strong effect the transformation has: The arcsinh-transformed data shows clear separation between assay types (right), something which is not present with the raw data (left), highlighting arcsinh-transform's benefit.

Why can we not compare Enformer Celltyping to Enformer?

Enformer Celltyping's uses Enformer as a pretrained model in our architecture but, it does not make sense to compare the predictive performance against Enformer. The main reason these models should not be compared is that they perform different, non-comparable tasks - Enformer predicts in held-out chromosomes and so does not have to extrapolate predictions to previously unseen cell types whereas Enformer Celltyping does on whole, held-out cell types. See Fig. 1a-b from Schreiber *et al.*,[3] for a visual representation of how these tasks differ and a detailed description of both.

To give more detail, Enformer is a basal or base model of Enformer Celltyping. We do this using transfer learning, a machine learning technique that allows us to repurpose pre-trained models on new task. However, this does not mean it is suitable to compare against the base model in either this new or the old task. To give an example, many models have used pre-trained image classification models trained on generic datasets such as Modified National Institute of Standards and Technology database (MNIST), which is pictures of handwritten digits, with fine-tuning to apply them to the medical domain. For example, for brain tumour classification from Magnetic resonance imaging (MRIs)[8]. Yet, comparing the MNIST, pretrained model to the brain tumour model on either task would not be beneficial. In the same way, we should not compare Enfomer to Enformer Celltyping.

Nevertheless despite this, and as a thought experiment, let's consider trying to compare the two models. If we were to compare Enformer to Enformer Celltyping, we should not do it based on either model's training set, as this would just produce inflated performance for both models. Moreover, we would be interested in the performance in functionally relevant regions (as we discuss for our other comparisons of Enformer Celltyping – Fig 4,5 and S7). Thus, the only option would be to test on the intersection of Enformer's test set and SCREEN functional regions[9] on cell types that Enformer trained on and that Enformer Celltyping did not train on, i.e. a blind test set for both. Overlapping SCREEN regions with Enformer's test set results in a total of just 152 promoter, proximal enhancer and distal enhancer regions. These are very small regions (on average ~143 base-pairs) and the overlapping base-pair positions were just 21,748 base-pairs total or 169 predicted positions since Enformer and Enformer Celltyping predict in 128 bp blocks. This equates to just 0.0001% of chromosome 1 alone. Given this is so small it isn't a representative test set and no insight should be taken from any results based on this. To give some comparison, Enformer tested on 1,937 test regions of predicted window size 114,688, i.e. 222,150,656 or 222 million base-pairs. Also, we tested Enformer Celltyping on 1,034,718 SCREEN positions i.e. 282,041,971 or 282 million base-pairs – approximately 13,000 times more positions. This issue is furthered if we calculate performance values at a chromosome level for the functional regions, to get multiple values to have a range in performance. Some of the chromosomes would have just single values and so would result in making computation of a correlation impossible.

Furthermore, comparing the models at the same positions would not even be possible with the above approach: The SCREEN locations that overlap Enformer would need to be found using SCREEN hg38. However, one would then need to map these regions to hg19 to use for Enformer Celltyping. There is little to say this mapping is accurate or that these resulting regions in hg19 are actually in the same functional regions in SCREEN.

Finally, Enformer predicts fold change and Enformer Celltyping predicts p-values. So the performance comparison wouldn't even be based on the same y values, just at similar positions for similarly named cells types.

From this explanation, we hope it is clear why Enformer Celltyping can not be compared against Enformer. We have however, compared Enformer Celltyping against the current best-in-class model for predicting histone mark signals in previously unseen cell types (i.e.

extrapolating predictions to previously unseen cell types) Epitome in Fig. 3a where our model showed notably strong performance. Moreover, we have compared Enformer Celltyping to Enformer for the genetic variant effect prediction task, a key task for genomic deep learning models, where they performed similarly (Fig.6a-b).

Supplementary References

1. Avsec, Ž. *et al.* Effective gene expression prediction from sequence by integrating long-range interactions. *Nat Methods* **18**, 1196–1203 (2021).
2. Kelley, D. R. Cross-species regulatory sequence activity prediction. *PLOS Computational Biology* **16**, e1008050 (2020).
3. Schreiber, J., Durham, T., Bilmes, J. & Noble, W. S. Avocado: a multi-scale deep tensor factorization method learns a latent representation of the human epigenome. *Genome Biology* **21**, 81 (2020).
4. Ernst, J. & Kellis, M. Large-scale imputation of epigenomic datasets for systematic annotation of diverse human tissues. *Nat Biotechnol* **33**, 364–376 (2015).
5. Durham, T. J., Libbrecht, M. W., Howbert, J. J., Bilmes, J. & Noble, W. S. PREDICTD PaRallel Epigenomics Data Imputation with Cloud-based Tensor Decomposition. *Nat Commun* **9**, 1402 (2018).
6. Kundaje, A. *et al.* Integrative analysis of 111 reference human epigenomes. *Nature* **518**, 317–330 (2015).
7. Ernst, J. & Kellis, M. Chromatin-state discovery and genome annotation with ChromHMM. *Nat Protoc* **12**, 2478–2492 (2017).
8. Mathivanan, S. K. *et al.* Employing deep learning and transfer learning for accurate brain tumor detection. *Sci Rep* **14**, 7232 (2024).
9. Moore, J. E. *et al.* Expanded encyclopaedias of DNA elements in the human and mouse genomes. *Nature* **583**, 699–710 (2020).
